# Fungi in the Marine Environment: Open Questions and Unsolved Problems

**DOI:** 10.1128/mBio.01189-18

**Published:** 2019-03-05

**Authors:** Anthony Amend, Gaetan Burgaud, Michael Cunliffe, Virginia P. Edgcomb, Cassandra L. Ettinger, M. H. Gutiérrez, Joseph Heitman, Erik F. Y. Hom, Giuseppe Ianiri, Adam C. Jones, Maiko Kagami, Kathryn T. Picard, C. Alisha Quandt, Seshagiri Raghukumar, Mertixell Riquelme, Jason Stajich, José Vargas-Muñiz, Allison K. Walker, Oded Yarden, Amy S. Gladfelter

**Affiliations:** a Department of Botany, University of Hawai’i at Manoa, Honolulu, Hawaii, USA; b Université de Brest, EA 3882, Laboratoire Universitaire de Biodiversité et Ecologie Microbienne, ESIAB, Technopôle Brest-Iroise, Plouzané, France; c Marine Biological Association of the United Kingdom, The Laboratory, Citadel Hill, Plymouth, United Kingdom; d Department of Geology and Geophysics, Woods Hole Oceanographic Institution, Woods Hole, Massachusetts, USA; e Genome Center, University of California, Davis, California, USA; f Departamento de Oceanografía, Centro de Investigación Oceanográfica COPAS Sur-Austral, Universidad de Concepción, Concepción, Chile; g Department of Molecular Genetics and Microbiology, Duke University Medical Center, Durham, North Carolina, USA; h Department of Biology, University of Mississippi, Oxford, Mississippi, USA; i Gordon and Betty Moore Foundation, Palo Alto, California, USA; j Graduate School of Environment and Information Sciences, Yokohama National University, Yokohama, Japan; k Department of Botany, National Museum of Natural History, Smithsonian Institution, Washington, DC, USA; l Ecology and Evolutionary Biology Department, University of Colorado, Boulder, Colorado, USA; m National Institute of Oceanography, Goa, India; n Department of Microbiology, Centro de Investigación Científica y Educación Superior de Ensenada (CICESE), Ensenada, Baja California, Mexico; o Department of Microbiology & Plant Pathology and Institute for Integrative Genome Biology, University of California-Riverside, Riverside, California, USA; p Department of Biology, University of North Carolina at Chapel Hill, Chapel Hill, North Carolina, USA; q Department of Biology, Acadia University, Wolfville, Nova Scotia, Canada; r Department of Plant Pathology and Microbiology, The Robert H. Smith Faculty of Agriculture, Food and Environment, The Hebrew University of Jerusalem, Rehovot, Israel; s Marine Biological Laboratory, Woods Hole, Massachusetts, USA; University of Texas Health Science Center at Houston

**Keywords:** mycology, chytrid, marine fungi, marine microbiology

## Abstract

Terrestrial fungi play critical roles in nutrient cycling and food webs and can shape macroorganism communities as parasites and mutualists. Although estimates for the number of fungal species on the planet range from 1.5 to over 5 million, likely fewer than 10% of fungi have been identified so far. To date, a relatively small percentage of described species are associated with marine environments, with ∼1,100 species retrieved exclusively from the marine environment. Nevertheless, fungi have been found in nearly every marine habitat explored, from the surface of the ocean to kilometers below ocean sediments. Fungi are hypothesized to contribute to phytoplankton population cycles and the biological carbon pump and are active in the chemistry of marine sediments. Many fungi have been identified as commensals or pathogens of marine animals (e.g., corals and sponges), plants, and algae. Despite their varied roles, remarkably little is known about the diversity of this major branch of eukaryotic life in marine ecosystems or their ecological functions. This perspective emerges from a Marine Fungi Workshop held in May 2018 at the Marine Biological Laboratory in Woods Hole, MA. We present the state of knowledge as well as the multitude of open questions regarding the diversity and function of fungi in the marine biosphere and geochemical cycles.

## Abstract (precis)

Terrestrial fungi play critical roles in nutrient cycling and food webs and can shape macroorganism communities as parasites and mutualists. Although estimates for the number of fungal species on the planet range from 1.5 to over 5 million, likely fewer than 10% of fungi have been identified so far.

## INTRODUCTION

The first records of marine fungi came from 19th century studies which utilized microscopy- and culture-dependent approaches, such as growing organisms on prepared media or on incubated samples collected from the marine environment (e.g., wood) (1–3). The relatively more recent application of culture-independent methods (e.g., DNA sequencing) has provided additional insights into marine fungal diversity (discussed further in the sections below) and has stimulated a new wave of interest into fungal functional roles in marine ecosystems, their potential applications in bioremediation, and as new sources of natural products of therapeutic value. One challenge plaguing the field of marine mycology has been in defining which fungi are truly “marine.” Many fungi that are found in the sea are also found in terrestrial environments, indicating the remarkably effective adaptive capabilities within the fungal kingdom. In this report, we discuss the known and postulated functional roles for fungi throughout the marine environment with an eye toward understanding the colonization of marine habitats by fungi and their contributions to the ecology of the sea. This perspective emerges from a Marine Fungi Workshop held in May 2018 at the Marine Biological Laboratory in Woods Hole, MA. Here, we present the state of knowledge as well as the multitude of open questions regarding the diversity and function of fungi in the marine biosphere and geochemical cycles. Opportunities and successes in developing new fungal model systems from the ocean are also considered.

## WHAT FUNGI ARE IN THE MARINE ENVIRONMENT?

Our understanding of marine fungal diversity and distributions is shaped, in large part, by the methods employed. Beginning from the first description of a “marine” fungus isolated from *Spartina* roots (4), most early efforts at describing marine fungal diversity focused on plant- and alga-associated species forming conspicuous macroscopic reproductive structures as well as those that were amenable to isolation in culture (3, 5). This focused sampling, which was predominantly nearshore, led to the perception of a marine mycobiota that was depauperate compared to terrestrial fungi and restricted largely to plant-based substrates. More recently, environmental DNA-based surveys have allowed a glimpse into inconspicuous and uncultivated marine fungal diversity in a broader suite of habitats, including those inside animal hosts, the water column, and ocean sediments, indicating a vast and phylogenetically diverse mycobiota likely differentiated by geography, substrate, and environmental conditions. Microscopy has enabled further insights into the identity and high prevalence of marine fungal pathogens on phytoplankton in aquatic systems (6, 7).

Fungal diversity has long been synonymous with terrestrial diversity. Marine fungi have largely been neglected, even though it is estimated that there are greater than 10,000 marine fungal species (5). Fungi have been found in nearly every marine habitat examined, including sediments (8), the water column (9), driftwood (10), sessile and mobile invertebrates (11), algae (12), and marine mammals (13), ranging in location from the deep sea all the way to surface waters. While a growing body of literature highlights that fungi are abundant, diverse, and widespread in marine habitats, these studies also emphasize how much work remains to be done. Undoubtedly, novel habitats, locations, and data sets will identify additional species occurrences.

To date, the vast majority of fungi identified from marine environments belong to the Ascomycota and Basidiomycota phyla (3), independent of whether culture, microscopy, or DNA-based methods are used (although see Richards et al. [14] for a different perspective using an alternative DNA-based approach). Marine and aquatic fungi also contain a wealth of novel and undescribed species at relatively high taxonomic ranks (15, 16). Particularly notable are a large number of species belonging to “early diverging lineages” such as the Chytridiomycota (chytrids), which tend to dominate nearshore and sediment samples (14, 17, 18). Much of the diversity known within these groups is almost entirely based on environmental sequencing data, the so-called dark matter fungi (19).

Novel species within lineages that are well-known from terrestrial habitats are frequently observed in studies of marine fungal diversity. Comeau et al. (18), for example, found high proportions of novel Chytridiomycota-like sequences from both arctic and temperate seawater. Other studies examining marine sediments, water columns, and invertebrate mycobiomes have identified new lineages of *Malassezia*, a genus generally considered dermatophytic due to its abundance in the skin of mammalian hosts and reliance on exogenous lipids (20). While some of these DNA sequences correspond to known and isolated species, evidence suggests a high diversity of novel species, although none have yet been isolated from marine habitats.

Researchers are often surprised to find that many fungi detected in marine environments are already well characterized from soil or plant habitats, even when those marine samples are collected from locations far from obvious terrestrial inputs. Tempting as it may be to interpret these data as evidence that a large proportion of marine fungi are metabolically inactive flotsam (as spores or relictual DNA), evidence suggests otherwise. Strong correlations with abiotic environmental conditions (8, 21) and gene expression data (22) suggest that at least some fungi display a truly amphibious ability (23). Furthermore, phylogenetic studies suggest that many obligately marine lineages recently transitioned from terrestrial ancestors (e.g., 24) and that such transitions to marine habitats have occurred multiple times. The ecological plasticity of fungi thus leads to some scientific soul searching for an operational definition of “marine” fungi. Pang et al. (25) have proposed the broad definition that a marine fungus is “any fungus that is recovered repeatedly from marine habitats and: 1) is able to grow and/or sporulate (on substrata) in marine environments; 2) forms symbiotic relationships with other marine organisms; or 3) is shown to adapt and evolve at the genetic level or be metabolically active in marine environments.”

## WHAT ARE THE CHALLENGES IN CHARACTERIZING MARINE MYCOBIOMES?

As is the case with many fields focused on environmental microbes, the shifting emphasis from cultivation-based studies (e.g., 26) to environmental DNA-based surveys (e.g., 27) has contributed significantly to our understanding of marine fungal diversity and distributions, but it has also led to unanticipated challenges that have hampered progress.

First, amplicon sequencing based on the fungal ITS rDNA region (the accepted fungal barcode, see reference 28) readily coamplifies other eukaryotes such as gelatinous zooplankton and invertebrate or plant hosts. Compounding this issue, these eukaryotes typically dominate marine environmental metagenomic sequence data, resulting in limited representation by marine fungi. The ITS rDNA region primers were designed using sequence alignments from largely terrestrial representatives and are greatly biased toward terrestrial Dikarya (Basidiomycota and Ascomycota), resulting in poor representation of other fungal phyla known to occupy marine habitats. This has led marine mycologists to employ an unusually high number of primers and genomic regions (29), making large-scale data syntheses problematic. Also problematic is that metagenome sequencing and amplicon-based methods alone are unable to distinguish metabolically inactive fungi from true marine fungi *viz.* Pang et al.’s postulates, much less enable a realistic interpretation of how such organisms contribute to ecosystem processes and host health. Thus, additional lines of evidence are needed to capture and characterize key fungal players in marine ecosystems.

While playing catch-up to other marine microbial fields may seem an unenviable position in which to be, marine mycologists hope to borrow from the best practices of the *Bacteria*, *Archaea*, virus, and protist communities to establish a vigorous and thriving framework documenting the diversity and distribution of fungi in the world’s oceans. Here, we identify three main objectives that we anticipate will help us to achieve these goals. First, we hope to establish a standardized set of sampling and processing protocols (see protocols for the Marine Fungi group), primers, and metadata so that future efforts might be compared across systems, recognizing that these might not extend naturally from terrestrial precursors. Continued cooperation, collaboration, and communication among marine mycologists and researchers in related fields will help achieve comparable research outputs. Second, we hope to establish and implement a global scale survey (akin to IcoMM [30] or TARA [31]) from which diversity hot spots and research priorities might be established. This might be partially achieved via “citizen science” efforts, via dedicated cruise and sampling efforts, or by revisiting existing samples or even data sets with methods that capture fungal diversity. Third, we hope to populate sequence databases, culture collections, and genomic resources with samples from marine origins. Few of the type specimens described by the prolific marine mycologists Jan and Erika Kohlmeyer (3), for example, have DNA sequence data deposited in public repositories, and there is no central, dedicated or publicly accessible collection of marine isolates anywhere in the world. By achieving these goals, we hope to gain insight into the diversity of these often overlooked ecosystem engineers in order to determine their unique contributions to marine ecosystems.

## HOW DO FUNGI INTERACT WITH THE MARINE BIOSPHERE?

**FIG 1:**
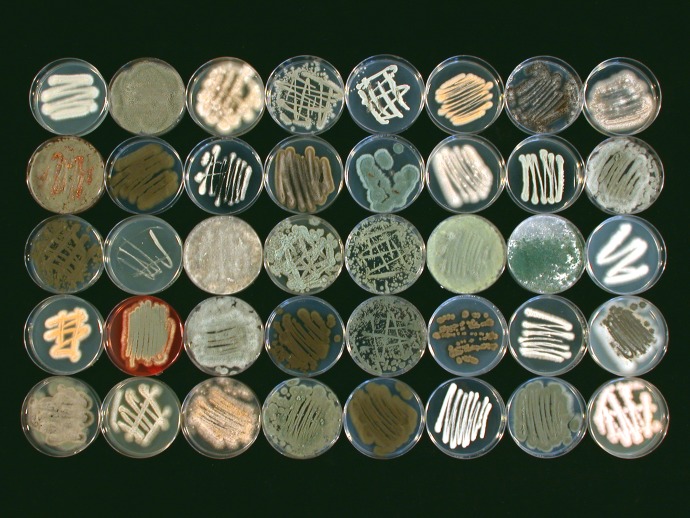
Morphological diversity of fungi collected from a biotic host. Fungal collection isolated from a marine sponge, Ircinia variabilis (formerly *Psammocinia* sp.). For details, see Paz et al. (35).

Considerable attention has been given to the analysis of fungus-biota interactions in terrestrial systems, but much less is known about such interactions in aquatic, particularly marine, environments. One of the earliest reports on algal parasitism by a marine fungus was documented 125 years ago (32). Since then, evidence for the presence of fungi in association with prokaryotes (bacteria and archaea), plants, and animal life forms has expanded, initially based largely on the pioneering work of the groups of Kohlmeyer and Jones (3, 5). Growing attention has been directed at determining the presence and prevalence of fungal species in association with other marine organisms (33–35) (Fig. 1). Based on our current knowledge, representatives spanning all known fungal phyla appear to associate with almost every marine organism studied thus far (11, 36).

Studies have also explored the effects of environmental conditions or the physiological state of the nonfungal (host) partner on fungal communities (22, 37, 38). The nature of these interactions remains unclear, although as in terrestrial systems, extracellular enzyme activities and secondary metabolite production might play significant roles in interactions of fungi with marine hosts (39). Fungal antibiotics directly influence the composition of marine bacterial communities (40) and thus, indirectly, the myriad processes of the hosts and ecosystems that depend on these communities.

**FIG 2:**
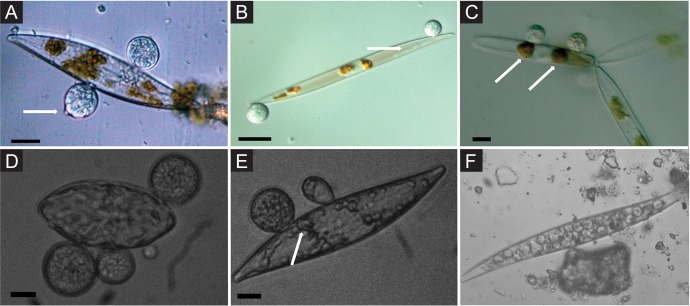
Chytrid parasites of marine diatoms. (A) Chytrid sporangia on *Pleurosigma* sp. The white arrow indicates the operculate discharge pore. (B) Rhizoids (white arrow) extending into diatom host. (C) Chlorophyll aggregates localized to infection sites (white arrows). (D and E) Single hosts bearing multiple zoosporangia at different stages of development. The white arrow in panel E highlights branching rhizoids. (F) Endobiotic chytrid-like sporangia within diatom frustule. Bars = 10 μm. Adapted from Hassett and Gradinger (43) with permission.

Chytrid associations with phytoplankton are one of the most notable examples of fungal pathogenicity in aquatic environments (Fig. 2). In freshwater systems, these fungi play a critical role in nutrient dynamics by infecting phytoplankton and making them more susceptible to predation by zooplankton. The handful of studies examining fungi in the open-ocean and coastal upwelling ecosystems demonstrate a positive correlation between phytoplankton and fungal abundance (41, 42). Fungal biomass typically lags behind that of phytoplankton by ∼1 month, typical of density-dependent pathogen-host interaction dynamics (41). Similar dynamics are observed in polar sea ice, where chytrid parasite abundance tracks that of diatom hosts, a relationship that is magnified when ice algae experience environmental stress (43). Associations with nonchytrid fungi have been shown to elicit defensive responses in corals (44–46) and lead to parasitism of the sea fan Gorgonia ventalina by Aspergillus sydowii (47), a fungal species known to bloom in coastal waters and impact the dinoflagellate symbiont *Symbiodinium* (48).

A second prominent fungus-host association has emerged within the genus *Malassezia*. These lipophilic fungi are nearly always detected in marine habitats when using DNA-based techniques (20), although their ecological functions remain unclear. This genus is related to known plant-pathogenic fungi and is also often found associated with human skin microbiota, where it thrives on lipid-rich sebaceous gland secretions (49). *Malassezia* produces a protease that exerts hydrolytic activity on the biofilm of the human bacterial pathogen Staphylococcus aureus (50). Could *Malassezia* (and other fungi) play similar or additional roles for marine hosts? Given recent discoveries of the importance of lipid transfer between arbuscular mycorrhizal fungi and plants (51), are lipids likely to be critical components of marine fungus-host associations as well? The chemical “dialogue” underlying marine fungus-host interactions is largely uncharted, although recent studies have shown marine fungi to be rich sources of novel biosynthetic clusters and secondary metabolites (52, 53).

Despite little data regarding the nature of marine fungal biotic interactions, studies demonstrating host specificity, coevolution, or phylosymbiosis *sensu* (54) indicate that at least some of these symbioses are strongly determined by the host and not merely stochastic associations. For example, fungal communities associated with two cooccurring Hawaiian marine sponges were significantly partitioned by host identity and differed from fungi in the surrounding water column (55). Similar patterns of host specificity have been observed in fungi associated with seagrass leaves (12), in mycorrhizae-like associations in seagrass roots (56), mesophotic macroalgae (12), and in scleractinian corals (57).

Thus far, attempts at understanding the function of fungi in marine habitats have adhered to concepts developed for the analysis of their terrestrial counterparts. This includes emphasizing fungal traits in relation to potential lifestyles (e.g., commensalism, pathogenesis, opportunism). However, is such a framework adequate/appropriate for interactions in marine environments? Significant efforts have attempted to link fungal presence/activity with diseases and syndromes (58), and examples of mutualistic interactions have been identified (59–63). Would alternative means for describing these interactions be appropriate? While studying fungal pathogenesis of animal systems, Casadevall and Pirofski (64) suggested that rather than focus on the processes leading to host damage (as portrayed in part by the lifestyles of the fungal partners), perhaps the damage/benefit conferred to the host would be a better measurable outcome of host-fungus interaction. Such a metric would describe the alteration of the physiological condition of the host as it occurs following the fungal challenge together with the environmental effects that contribute to or affect the interaction. For example, they suggest adopting a “damage-response curve” as a means of quantifying interaction outcomes ranging from beneficial to pathological. As methods for evaluating the physiological state of marine macrobiota progress, measuring damage-response curves may become feasible for assessing fungal interactions and their impact on hosts. Environmental and geographical variations influencing hosts would need to be accounted for, however, which may be more challenging for hosts from a marine ecosystem.

Interactions between fungi and other marine biota are likely to have significant implications that extend beyond the individual host or local community. Chytrids that parasitize phytoplankton such as colonial and filamentous cyanobacteria and diatoms provide a good example through a modified trophic linkage known as the mycoloop (7, 65, 66). Parasitic chytrids modify the amount and composition of DOC released from phytoplankton, which affect bacterial community structures (67). Could other fungi have similar trophic bridging, complementary, or competing roles? Molecular-based inventories of marine fungal diversity have recovered novel OTUs (operational taxonomic units) allied to known plant- and animal-associated lineages from seawater and marine sediments (9, 14, 15, 68, 69), suggesting that the myriad symbiotic interactions observed in terrestrial fungi—and their critical roles in ecosystem functioning—are likely present, or at least have correlates, in marine habitats as well.

## HOW DO FUNGI INFLUENCE MARINE BIOGEOCHEMICAL CYCLES?

The number of studies that directly address how marine fungi influence the geochemistry of the oceans are scarce relative to other microbial groups, although growing evidence strongly suggests that fungi impact biogeochemical cycles in multiple and complex ways. For accurate climate change modeling and remediation, a deeper understanding of how fungi control major nutrient fluxes in time and space is essential, and it is critical to develop new ways to measure the activity of fungi *in situ* and not simply report their presence. Below, we discuss the role of fungi in the marine carbon cycle (MCC) as well as their contributions to the degradation of anthropogenic hydrocarbons such as oil and plastics.

The MCC is a vital earth system process driven by photosynthetic phytoplankton in the surface euphotic zone, converting dissolved inorganic carbon to organic matter and producing oxygen. Phytoplankton and the organic matter they produce are the foundations of marine food webs, supporting heterotrophic bacteria, protists, viruses, zooplankton, and ultimately, higher trophic organisms that include fish and marine mammals (70). A proportion of the organic matter produced in the euphotic zone sinks as “marine snow” through the mesopelagic and bathypelagic zones via the biological carbon pump, removing the sequestered carbon from surface waters and transferring it to the deep ocean (71). A key open question is the degree to which fungi contribute to this biological carbon pump both at this time and in the context of climate change.

Quantifying microbial biomass, both standing stocks and turnover rates, is essential for our understanding of the functional roles that microbes fulfil in marine ecosystems. So far, only a few studies have assessed fungal biomass in the marine water column (6, 9, 72, 73). Gutiérrez et al. (72) used calcofluor white staining and epifluorescence microscopy of chitin-containing hyphae to determine fungal biomass in the coastal upwelling ecosystem of the Humboldt Current System off Chile. Fungal biomass in the water column decreased monotonically with depth, had a seasonal cycle, was comparable to prokaryote biomass, and coincided with increases in phytoplankton biomass. These findings challenge the current view that bacteria and archaea are the principal contributors of heterotrophic microbial biomass in the surface ocean. The significance of fungal biomass in marine ecosystem carbon flux models remains a pressing open question.

Measurements of substantial fungal biomass are not unique to the Pacific Ocean or shallow waters but have also been made in relation to the deep Atlantic Ocean. Using tyramide signal amplification CARD-FISH, fungi were found to be a dominant fraction of bathypelagic marine snow particles collected from the North Atlantic Ocean (73), suggesting that fungi contribute to the transport of carbon and other nutrients by marine aggregates in the deep ocean. In the coastal waters of the Western English Channel, fungus-specific quantitative PCR (qPCR) was used as a proxy for biomass and revealed that changes in fungal density are linked to a range of physicochemical drivers, including increased particulate organic carbon (POC) availability and salinity fluctuations (9). Turnover rates of marine fungal biomass are not yet known; however, molecular analyses of zooplankton gut contents indicate that fungi can form a substantial proportion of their diet (74, 75). Collectively, these biomass studies support the hypothesis that fungi influence the flux of biomass-associated carbon in the oceans globally.

**FIG 3:**
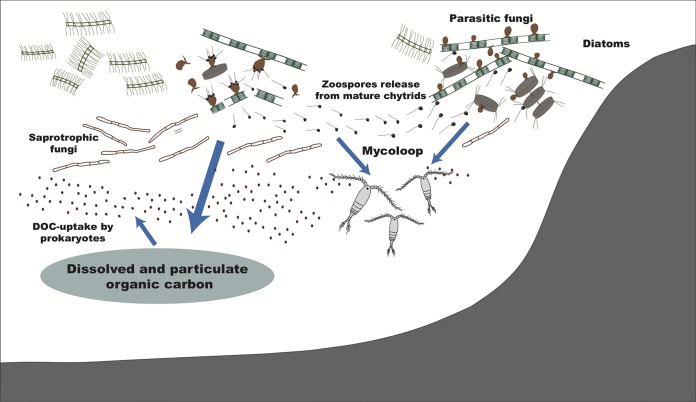
Roles of fungi in the marine carbon cycle by processing phytoplankton-derived organic matter. Parasitic fungi, as well as saprotrophic fungi, directly assimilate phytoplankton organic carbon. By releasing zoospores, the fungi bridge the trophic linkage to zooplankton, known as the mycoloop. By modifying the particulate and dissolved organic carbon, they can affect bacteria and the microbial loop. These processes may modify marine snow chemical composition and the subsequent functioning of the biological carbon pump. Modified from Gutierrez et al. (41) with permission.

Marine phytoplankton can be infected by not just Chytridiomycota but also Cryptomycota and Aphelida (76, 77). Thus, early diverging zoosporic fungi may directly impact the keystone drivers of the oceanic carbon cycle (9, 16, 18, 41–43, 78). Although some marine phytoplankton are evidently infected by chytrids, the impacts of these infections on phytoplankton ecology and the MCC need more investigation. If these relationships are analogous to freshwater chytrid-phytoplankton interactions, then the impacts on oceanic biogeochemical cycles could be significant, including the release of particulate and dissolved organic carbon, the modification of marine snow chemical composition, and the subsequent functioning of the biological carbon pump (28). Therefore, there is reason to suspect a marine version of the mycoloop exists and could be a critical element of global carbon cycling (Fig. 3).

A key challenge is to identify not simply the presence of fungi but also their activity. Some fungi in the water column appear to have a role in the MCC by processing phytoplankton-derived organic matter (72, 79). This has been demonstrated by fractionation of extracellular enzymes and assessing their activity through incubation with fluorogenic substrates. Activity appears to be associated with periods of high phytoplankton and fungal biomass (6). An alternative approach for evaluating activity focuses on measuring the incorporation of carbon from other marine organisms into fungi. Using DNA stable isotope probing (DNA-SIP) with ^13^C-labeled diatom-derived polysaccharides, specific fungal taxa, including the hyphomycete *Cladosporium*, directly assimilate phytoplankton organic carbon, and it is clear that *Cladosporium* secretes the extracellular enzyme glucan 1,3,-β-glucosidase that can be used to digest phytoplankton-derived organic matter (79). *Cladosporium* abundance in particular correlates with increased abundances of specific diatom species and in the deep chlorophyll maxima regions of the oceans where phytoplankton biomass can be highest (79).

Fungal activity is not limited to the water column and may even be more prominent in ocean sediments. Using a range of subsurface sediment samples collected from up to 48 m below the sea floor (mbsf), rRNA-based assessments of active eukaryote biomass show that fungi can dominate within these ecosystems, particularly in sediments containing high proportions of organic carbon (8). Metatranscriptome analysis of very deep (i.e., up to 159 mbsf) sediment samples of the Peru Margin revealed fungi actively engaged in processing a range of different organic matter types, including lipids, proteins, and carbohydrates via specific hydrolases (80). Deeper down in Canterbury Basin subsurface sediments (up to 350 mbsf), fungal gene expression was associated with growth, division, and sporulation, catalytic activities, and the synthesis of antimicrobial products (81). These studies indicate nutrient recycling and cross-feeding interactions between fungi and other microbial communities, as well as competitive interactions through the synthesis of antimicrobial and antibiofilm compounds.

The deep oceanic crust is one of the last great frontiers for biological exploration on earth. Our understanding of the habitability and biological diversity in this environment is still in its infancy, and deep ocean crust fungi may play important roles as symbionts with chemoautotrophic prokaryotes (82), in decomposing organic matter (83), in mineral weathering (82), and in manganese (84) and arsenic cycling (85). There is an emerging hypothesis that fungal hydrogenosome-based anaerobic metabolism supplies molecular hydrogen to methanogens and other hydrogen-consuming archaea in deep igneous oceanic crusts (86), highlighting complex interactions between marine fungi and other microbial communities yet to be fully characterized.

In addition to natural carbon cycles, fungi appear to play fundamental roles in cycling anthropogenic sources of carbon. In assessing the microbially diverse populations of coastal sediments in the Gulf of Mexico before and after the Deepwater Horizon (DH) oil spill, fungi were found to dominate benthic communities impacted by oil and included taxa known to degrade hydrocarbons (87). After the DH oil spill, CONACYT (the Mexican Science and Technology Council) and the Mexican Secretary of Energy funded the Gulf of Mexico research consortium (CIGoM) (https://cigom.org/), established among multiple Mexican research centers and universities and led by a group of researchers at CICESE (https://www.cicese.edu.mx/). The main goal of the consortium was to establish the baseline of the Mexican Exclusive Economic Zone (EEZ) of the Gulf of Mexico for oceanographic, biogeochemical, ecological, and biological variables, to evaluate the potential damage that could occur in the event of oil spills, and to design mitigation strategies. One of the subprojects of this consortium analyzed by ITS-based amplicon sequencing the benthic mycobiota diversity of deep-sea sediments and also obtained fungal isolates to evaluate their ability to degrade hydrocarbons (M. Riquelme, unpublished data). In oil-polluted sediments, fungi are likely primary degraders of high-molecular-weight hydrocarbons via secreted extracellular enzymes and work synergistically with oil-degrading bacteria (88). Fungi are thought to have a relatively high tolerance to hydrocarbons (89), and more than 100 genera are known to play important roles in biodegradation of hydrocarbons in soils and sediments (90–96). Filamentous fungi such as *Cladosporium* and *Aspergillus* are among those known to participate in aliphatic hydrocarbon degradation, and the genera *Cunninghamella, Penicillium, Fusarium*, *Mucor*, and *Aspergillus* are among those known to take part in the degradation of aromatic hydrocarbons (89, 97, 98). While most filamentous fungi investigated thus far are unable to fully mineralize aromatic hydrocarbons, fungi may participate with other microorganisms in their degradation (99).

Plastics have become the most common form of waste in the environment and represent a major and growing environmental and global threat, with an annual plastic waste input from land into the ocean of 4.8 to 12.7 million metric tons (100). Recent records of deep-sea plastic pollution have also highlighted the ubiquitous nature of plastics even at depths of >6,000 m (101). While several studies highlighted numerous bacterial OTUs representing putative hitchhikers (102–106), few studies have so far specifically targeted microeukaryotic communities, and more precisely fungal communities, associated with plastic debris. Metabarcoding approaches have revealed different microeukaryotic communities associated with marine plastic waste, mostly polyethylene terephthalate (PET), polyethylene (PE), and polypropylene (PP), including diatoms, Phaeophyceaea, Chlorophyta, and fungi as dominant taxa (104, 106). To date, only one study has highlighted the ability of a coastal marine fungus, Zalerion maritimum, to degrade PE when cultured on a minimal medium (107). Despite their apparent ecological importance, marine fungal communities associated with marine debris have been largely overlooked, but concept studies are paving the way to better understand their abundance, distribution patterns, diversity, and ability to degrade plastic polymers.

Based on studies performed so far, it is clear that fungi are a thriving, abundant, active, and functioning component of the oceans, from surface sunlit waters to deep subsurface sediments and crusts, influencing marine biogeochemical cycles in multiple ways. We are in exciting times for marine fungal functional biology and ecology, and studies of these communities will very likely force us to rethink global biogeochemical cycles.

## ESTABLISHING MARINE FUNGAL MODEL SYSTEMS

In addition to the need for many more careful studies of fungi in the biological and geological context of the ocean, there is an equally critical need to cultivate and manipulate fungi in the lab to gain a mechanistic understanding of their evolution and ecological function. We lack a tool kit for molecular manipulations necessary for investigating the cellular biology and genetics of these marine fungi from the scale of single cells to complex multikingdom interactions. A primary challenge lies in the selection and definition of fungal model systems representing the marine environment, which will be context and sampling dependent. Within our current understanding of marine mycology, a model system could be defined by a single fungal species or whole communities contained within a given habitat or ecosystem (e.g., marine flora, marine sponges, coral and other invertebrates, and/or marine vertebrates). Model system development would ideally be informed by both ecological and evolutionary context, and selected fungal strains should be transformable and easily manipulated. Below, we discuss criteria for model system selection, challenges facing this field, success stories in model development, and future targets.

The ideal characteristics of a model system often depend on the questions posed (108). However, based on modern tools and techniques, some attributes are easily identified as most desirable in a marine fungus model. These could include the ability of the fungus to grow axenically in culture, with the potential for genetic transformation, a high-quality annotated reference genome, availability of multiple isolates (knowledge of genetic diversity), and the existence of known, closely related, terrestrial taxa, which may help illuminate specific adaptations to the marine environment. Establishing models that represent the breadth of fungal diversity (not just the Dikarya) would also be ideal.

Alternatively, models could be developed based on a particular marine host rather than focusing on a specific fungal taxon. Advantages to this approach include a clear target for sampling and methodological development, as well as a more holistic understanding of marine host mycobiota over time and space, i.e., studying fungi consistently found associated with a given host versus those that might be more transient or opportunistic in nature. Some marine animals are amenable to growth in the laboratory, making it possible to perform comparative and experimental microbiome studies under more natural culture-based growth conditions.

Species with dependency on marine conditions should also be considered as potential models. For example, in Acremonium fuci, conidial germination occurs only in the presence of tissue from its seaweed host, Fucus serratus, or aqueous tissue homogenates (109). Many marine fungi grow well in high-salt conditions, but Candida oceani seems obligately marine, as it displays optimal growth at 3% sea salt (110). Other potential model organisms are those impacted when grown under marine conditions, such as a marine strain of Candida viswanathii exhibiting filamentous morphology under elevated hydrostatic pressure (111) or marine *Aspergillus* sp. with abnormal morphology at 20 MPa (112).

There are several success stories with regard to establishing new marine fungal model systems. Considerable work has been done developing the cosmopolitan, arenicolous marine fungus Corollospora maritima in the class Sordariomycetes (Ascomycota). In addition to a publicly available genome (https://genome.jgi.doe.gov/Corma2/Corma2.home.html), there has been transcriptome analysis under freshwater versus saltwater conditions (23), as well as population genetics and structure studies of this species (113). Moreover, C. maritima is easy to find, collect, and grow axenically in the laboratory. A closely related soil-inhabiting fungus, Microascus trigonosporus, also has a genome available (https://genome.jgi.doe.gov/Mictr1/Mictr1.home.html), and research is ongoing to develop this strain as a model for comparison with *C. maritima* (J. Spatafora et al., unpublished data).

With respect to model marine fungal hosts, there has been success with studying fungi associated with marine sponges and corals using both culture-based (34, 35) and culture-independent (37, 55) techniques. Recent studies have reported new fungal species from sponges (114) and examined how environmental factors impact fungal communities in coral hosts (37, 57, 115). Other potential fungal models that are in various stages of development include several in the Ascomycota, including Phaeotheca salicorniae, Knufia petricola, and Hortaea werneckii (A. Gladfelter et al*.,* unpublished data), and multiple ongoing genome sequencing projects of marine fungi via the 1000 Fungal Genomes Project at the Department of Energy’s Joint Genome Institute. Up-to-date curation of new species and literature can be found at http://www.marinefungi.org/.

There are, however, a variety of challenges in establishing new marine fungal models. The lack of shared repositories for culturing/isolation protocols, access to well-validated, publicly available isolates, and a lack of available deep RNAseq or proteomic data sets are current limitations in establishing model systems for marine fungi. A major challenge for modern marine mycology, as with microbiology, is the inability to easily culture the majority of microbially diverse populations revealed through metagenomic studies. A noteworthy example is the absence of a cultured marine isolate of *Malassezia* (mentioned above). While contamination of some marine samples with DNA or cells of a ubiquitous commensal and pathogen of human skin is possible in some examples, sequences related to but not identical to known *Malassezia* species suggests that at least some marine DNA sequences represent unsampled taxa. Although *Malassezia*-like yeast DNA is generally ubiquitous among marine habitats (14, 49), repeated efforts to isolate marine *Malassezia*-like yeast have been unsuccessful (unpublished studies by the authors of this article). This might reflect the fact that marine *Malassezia*-like yeasts are phylogenetically related to the human skin inhabitant Malassezia restricta, which is more fastidious to grow axenically than other *Malassezia*. Nevertheless, due to its ubiquitous nature in marine environments and its medical importance, *Malassezia* is a relevant target fungus for model development. Terrestrial *Malassezia* have compact genomes (∼7 to 9 Mb) that have undergone extensive genome rearrangements and gene loss/gain events (49). Although yet to be performed, a comparison of marine and terrestrial *Malassezia* may shed light on relevant mechanisms of genome evolution and adaptation, as well as the genetic arsenal required to colonize distinct ecological niches. We face additional challenges when trying to choose model fungi associated with specific hosts within the marine environment. This is true both from the viewpoint of some of the fungi (e.g., obligates, which may be difficult to study outside their hosts) as well as some of the hosts, perhaps most notably sponges which may be difficult to maintain in a laboratory setting (116). Thus, challenges exist at the level of availability of comparative models, culturing, and lack of understanding of fungus-host relationships. Nevertheless, we view each of these challenges as surmountable with targeted efforts.

What would a successful pipeline for establishing new marine fungal models look like? Collaboration, sharing methods and data, and frequent communication have been shown to be highly successful in establishing new experimental model systems (117). Within marine mycology, it will be critical to bridge the gaps between metagenomic surveys of marine environments and the existing, scattered collections and knowledge bases of marine fungi worldwide, bringing the latter into the genomic era. Several community-level initiatives could help establish new model systems in marine fungi including establishing a cookbook of fungal media that includes a panel of conditions that is open access and continuously updated and includes practical developments, successes, failures, and improvements on methods. For this purpose, the online protocol repository protocols.io is ideal, and a Marine Fungi group is established on this site. As a community, targets for model system development based on ecological and phylogenetic context and tractability should be prioritized. Finally, where possible, a transformation pipeline should be created with guidelines for marker selection, mechanisms of DNA transfer (e.g., electroporation, conjugation by Escherichia coli or *Agrobacterium*), and verification.

How do we advance our understanding of the nature and significance of fungal interactions within the marine environment? It is clearly essential to determine which fungal species are involved, and as already stated, mounting evidence suggests that many of the observed species have close terrestrial counterparts, raising interesting ecological questions regarding their abilities to adapt to marine conditions that need to be demonstrated using integrated approaches (81, 118). Significant efforts should be invested in both metagenomic/transcriptomic-based analyses as well as diversification in culturing approaches. Another important approach for gaining a better understanding of the nature of interactions between fungi and their hosts is to discover and develop amenable model host systems that permit experimental manipulations to determine the outcome of general and specific changes in the mycobiome. Aptaisia pallida is a proposed Cnidarian host model, and transcriptomic evidence suggests that fungi are both present and active in this anemone (119). As we identify and study specific marine fungal models, we advocate pursuing commensurate studies focused on their interactions with hosts and environments. Such studies would provide useful contextual knowledge not only for elucidating the potentially unique biology of these fungi but may help toward developing practical methods for experimental manipulation. For example, a deeper understanding of the life history traits and associations of potentially novel fungi associated with coral hosts (57) may help not only in understanding the nature of disease but may also yield nutritional insights for developing cultivation methods and/or facilitate the development of husbandry techniques in the laboratory.

## SUMMARY AND PERSPECTIVES

This report is a synopsis of discussions at the Woods Hole Marine Fungi Workshop in May 2018 and is intended to catalyze future work toward understanding the identity and function of fungi in marine environments. To facilitate and accelerate discovery, we have created a group on protocols.io on Marine Fungi where protocols and practical discussions can be shared, and we encourage people interested in this field to join this space. Marine fungal diversity estimates are kept and updated at http://www.marinefungi.org. There is clear and ample evidence that fungi shape both biological and geochemical cycles at all levels of the ocean ecosystem, but there are vast gaps in our mechanistic understanding of fungal ecosystem function. We suggest that system-scale approaches are needed to truly understand how fungi participate in different ecosystems within the ocean and advocate that cutting-edge tools need to be developed to detect fungal activity. This is an area rich with problems whose solutions will likely have profound implications for understanding and reacting to global climate change.
